# Circadian clock-gut microbiota axis in pulmonary hypertension: linking gut–lung crosstalk, immune timing and vascular remodeling

**DOI:** 10.3389/fphys.2026.1895249

**Published:** 2026-07-27

**Authors:** Ruixian Wu, Shuang Fu, Yiwen Zhang, Yuanyuan Dong, Jian Wang

**Affiliations:** 1 Guangzhou Laboratory, Guangzhou International Bio Island, Guangzhou, Guangdong, China; 2 State Key Laboratory of Respiratory Diseases, National Center for Respiratory Medicine, Guangzhou Institute of Respiratory Health, the First Affiliated Hospital of Guangzhou Medical University, Guangzhou, Guangdong, China; 3 Guangdong Key Laboratory of Vascular Diseases, National Clinical Research Center for Respiratory Diseases, Guangzhou Institute of Respiratory Health, the First Affiliated Hospital of Guangzhou Medical University, Guangzhou, Guangdong, China

**Keywords:** chronopharmacology, circadian rhythm, gut microbiota, gut-lung axis, immune inflammation, microbial metabolites, pulmonary hypertension

## Abstract

Pulmonary hypertension (PH) is a severe disorder characterized by progressive pulmonary vascular remodeling, increased pulmonary arterial pressure, and right heart failure. Beyond local abnormalities within the pulmonary vasculature, growing evidence suggests that PH is shaped by systemic inflammation, metabolic dysregulation, gut microbiota imbalance, and disruption of host circadian organization. In this review, we examine the circadian clock-gut microbiota-gut-lung axis as a time-dependent regulatory network in PH. We focus on core clock gene dysfunction, impaired intestinal barrier integrity, altered microbial metabolites, temporal monocyte and macrophage trafficking, NLRP3 inflammasome activation, and the feedback imposed by chronic hypoxia. The evidence is not equally strong across these mechanisms. Gut dysbiosis, altered microbial metabolites, and inflammatory monocyte activation have relatively direct PH-related support, whereas bile acid rhythmicity, engineered chronobiotic bacteria, and nanoparticle-mediated butyrate delivery remain more speculative. We therefore propose a hypoxia-circadian rhythm-gut microbiota-pulmonary vascular remodeling model as a testable framework rather than a proven causal pathway. This distinction matters: the next step for the field is not simply to identify different microbes, but to determine whether gut-derived inflammatory and metabolic signals lose their timing in PH and whether this loss of timing contributes to vascular remodeling.

## 1 Introduction

**Figure 1:**
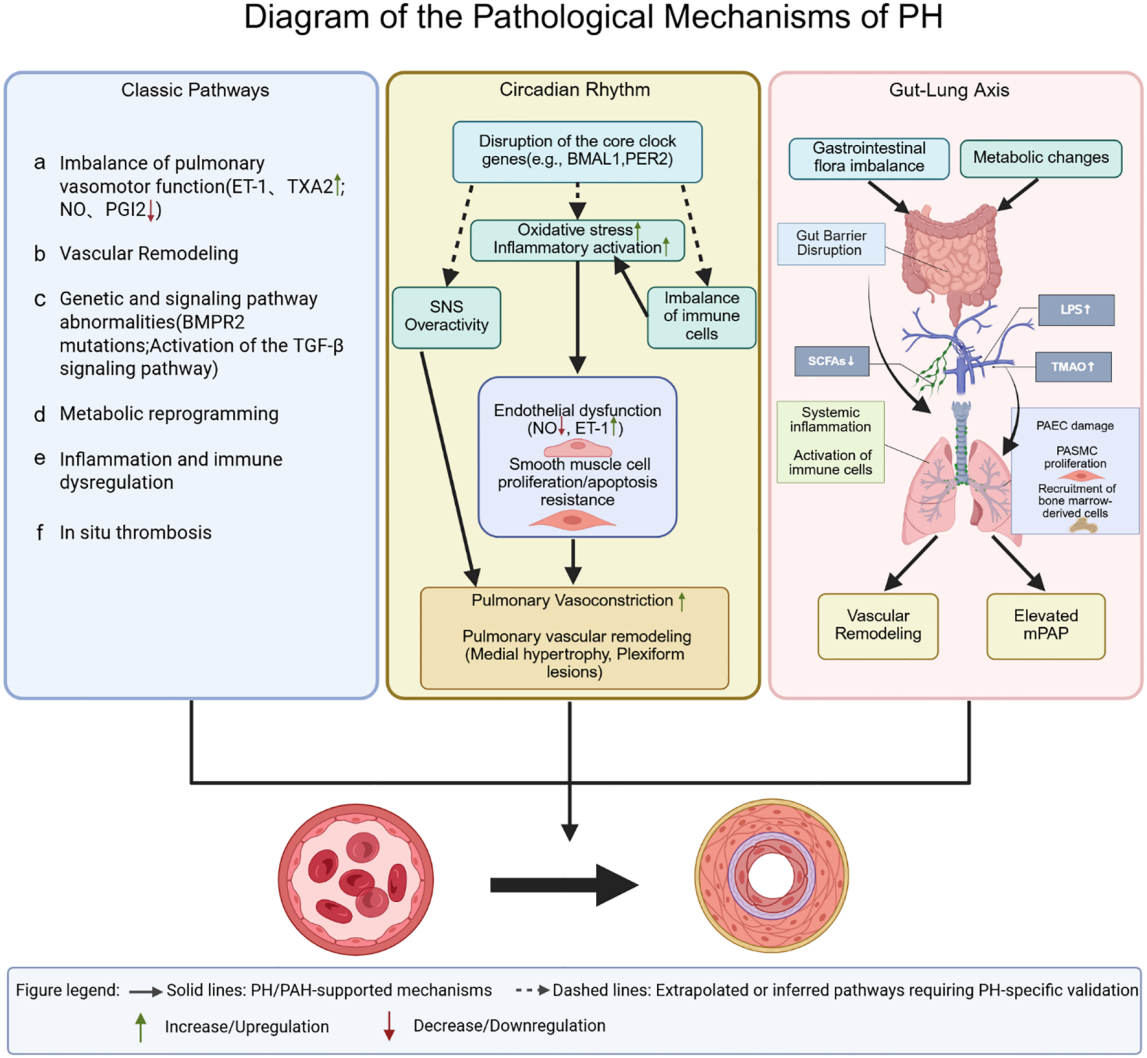
Pathophysiological mechanisms of PH driven by classic pathways, circadian rhythm disruption, and the gut-lung axis. The transition from health to disease involves three interconnected modules: (Left) Classic Pathways, (Middle) Circadian Rhythm and (Right) Gut-Lung Axis. Arrows indicate promotional pathways: ↑ (green) and ↓ (red) denote upregulation and downregulation, respectively. Created using BioRender.com.

PH is characterized by progressive pulmonary vascular occlusion, increased pulmonary vascular resistance, and eventual right heart failure. At the cellular level, the disease involves abnormal proliferation, migration, inflammatory activation, and apoptosis resistance of pulmonary arterial endothelial cells (PAECs) and pulmonary arterial smooth muscle cells (PASMCs). Targeted vasodilator therapies have improved outcomes for some patients, but they do not fully explain or reverse the broader disease process (Alsumali et al., 2025; Bertoletti et al., 2025). This gap has shifted attention toward mechanisms outside the vessel wall, including systemic inflammation, metabolic dysregulation, and inter-organ communication. The gut-lung axis sits at this intersection. It connects gut microbial composition, intestinal barrier integrity, circulating metabolites, and pulmonary immune tone (Maruyama et al., 2025, 2026). In PH patients and experimental PH models, studies have reported gut microbial dysbiosis, lower SCFA availability, increased TMAO, intestinal barrier impairment, and systemic low-grade inflammation (Chen et al., 2022; Cao et al., 2024; Prisco et al., 2025). These observations are important, but they are mostly static. They tell us what differs between groups, not when these differences appear or whether timing changes disease biology. We argue that the missing variable is biological time: gut-derived inflammatory and metabolic signals may be more pathogenic when they occur at a vulnerable circadian phase (**Figure 1**).

Circadian rhythms are endogenous oscillations of approximately 24 h that regulate cardiovascular function, immune responses, metabolic homeostasis, and barrier integrity, all of which are closely related to PH pathobiology (Zhen et al., 2023; Visniauskas et al., 2024). For gut-lung axis research, this adds more than a background variable. The host clock shapes feeding behavior, hormone release, intestinal motility, immune-cell abundance, and microbial metabolic output. Microbial products, including SCFAs, bile acids, tryptophan derivatives, and TMAO, feed back on clock-regulated inflammatory and metabolic pathways (Thaiss et al., 2016; Heddes et al., 2022). In PH, hypoxia, inflammation, and right heart dysfunction may disrupt this dialogue and establish a self-amplifying pathological circuit involving circadian misalignment, gut microbial dysbiosis, and pulmonary vascular remodeling (Manella et al., 2020; Sharma et al., 2020a; Abudukeremu et al., 2024). Because PH is a hemodynamic syndrome rather than a single disease entity, this review uses PH as an umbrella term but focuses primarily on WHO Group 1 pulmonary arterial hypertension and hypoxia-related experimental PH models, including chronic hypoxia and Sugen/hypoxia models. Evidence from CTEPH, PH associated with left heart disease, and non-PH inflammatory or metabolic diseases is explicitly identified when discussed.

This review is narrative and hypothesis-generating. We do not present circadian disruption or gut-lung axis dysfunction as established primary causes of PH. Instead, we use the available literature to build a mechanistic framework that can be tested with time-stratified clinical cohorts, interventional animal studies, and multi-omics designs.

## 2 Circadian rhythms and the gut-lung axis: bidirectional communication and network regulation

### 2.1 Core circadian clock system

**Figure 2:**
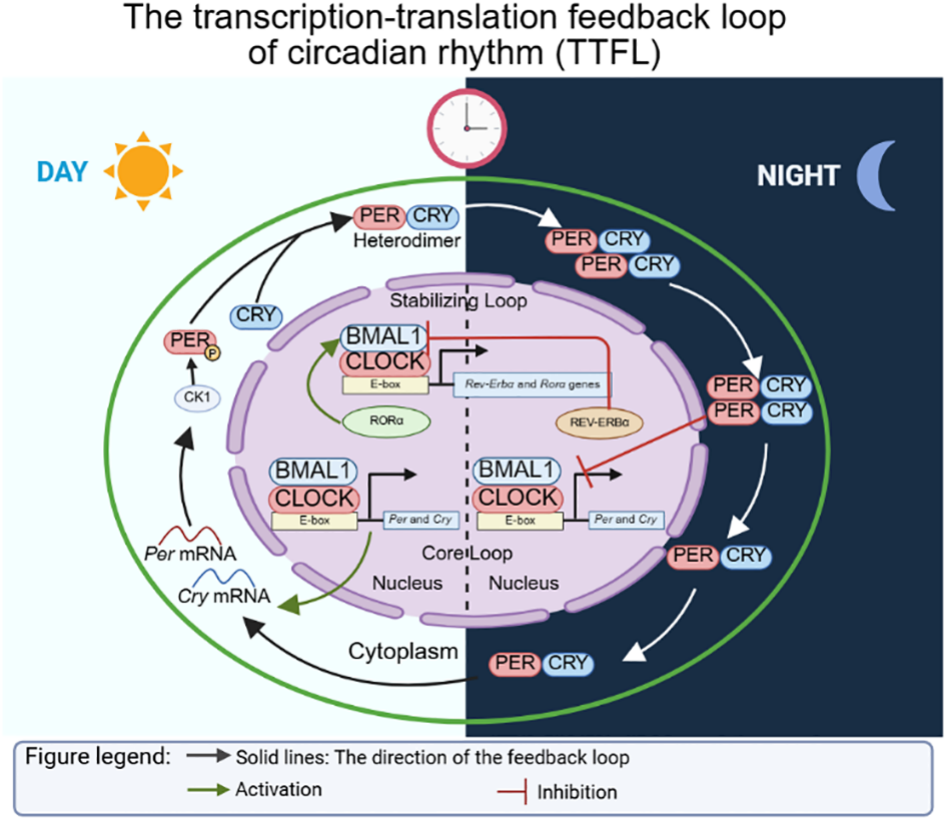
Molecular architecture of the circadian transcription-translation feedback loop (TTFL). The molecular clock operates via diurnal synchronized feedback loops: During the day, BMAL1/CLOCK heterodimers bind E-boxes to drive Per and Cry transcription. Post-translationally, PER is phosphorylated by CK1, forming stable PER/CRY complexes that translocate back into the nucleus at night to repress BMAL1/CLOCK activity. Concurrently, BMAL1/CLOCK regulates *Rev-Erbα* and *Rorα* expression. RORα and REV-ERBα proteins competitively bind the Bmal1 promoter to activate (green arrow) or repress (red bar) its transcription, ensuring robust ~24-h oscillations. Created using BioRender.com.

The core circadian clock system is a highly conserved transcription-translation feedback loop composed primarily of BMAL1, CLOCK, PER, CRY, REV-ERB, and ROR family clock genes and their protein products (Koike et al., 2012) (**Figure 2**). In mammals, the suprachiasmatic nucleus (SCN) of the hypothalamus functions as the central pacemaker that receives photic input and coordinates local clocks in peripheral tissues, including the lung, intestine, liver, and immune system, through neural, endocrine, and humoral pathways (Guo et al., 2005; Paul et al., 2020). But peripheral tissues are not passive recipients of SCN commands. Lung, intestine, liver, and immune cells each contain local clocks that regulate cell-cycle progression, energy metabolism, inflammatory tone, and barrier function. For PH, this distinction is central. A change in the SCN may influence autonomic and endocrine output, but local clocks in PAECs, PASMCs, macrophages, and intestinal epithelial cells are more likely to shape the immediate biology of vascular remodeling. Evidence for a central clock-autonomic nervous system-gut-lung communication pathway in PH remains limited. Thus, this network is best regarded as a plausible upstream regulatory system rather than a fully established pathogenic axis.

### 2.2 Gut-lung axis

The gut-lung axis is a functional framework describing bidirectional communication between the gastrointestinal tract and the respiratory system. Its major components include the gut microbiota, intestinal epithelial barrier, mucosal immune system, circulating metabolites, and neuroendocrine regulatory networks (Can et al., 2025; Huang et al., 2025; Maruyama et al., 2026). Gut microbes ferment dietary fiber to generate SCFAs, while bile acids and tryptophan derivatives are also transformed under microbial influence; these metabolites can be absorbed, metabolized by the liver, and distributed through the systemic circulation to distant organs (Lai et al., 2026). When the intestinal barrier is weakened, microbial-associated molecular patterns such as LPS can reach the circulation and promote low-grade inflammation, with downstream effects on pulmonary immune cells and vascular function (Burton et al., 2024; Chen et al., 2025b; Li et al., 2025b, 2025). In PH, this axis is supported by patient and animal-model data, but the findings are not perfectly aligned. Differences in disease stage, PH subtype, diet, medication exposure, sample source, and sequencing or metabolomics methods probably explain part of the heterogeneity. Thus, gut-lung communication should be viewed less as a single pathway and more as a set of interacting routes that may amplify disease in selected contexts.

### 2.3 Circadian regulation of intestinal homeostasis and microbial rhythmicity

Circadian rhythms regulate intestinal homeostasis through feeding-fasting cycles, hormonal secretion, body temperature, intestinal motility, and immune activity, thereby shaping diurnal fluctuations in gut microbial structure and metabolic function. Clock genes expressed in intestinal epithelial and immune cells regulate tight junction proteins, mucus secretion, antimicrobial peptide expression, and nutrient absorption, which collectively maintain intestinal barrier integrity (Guo et al., 2025; Zhao and Wu, 2025; Weng et al., 2026). Shift work, jet lag, irregular eating patterns, and sleep deprivation can weaken microbial rhythmicity, increase intestinal permeability, reduce beneficial commensals, and facilitate the expansion of potentially pathogenic taxa (Butler et al., 2025). In PH animal models, intestinal villus shortening, reduced goblet cell numbers, muscular layer thickening, and increased intestinal permeability have been observed, suggesting that circadian disruption may increase susceptibility to PH-related inflammation and metabolic abnormalities by weakening intestinal homeostasis and microbial rhythmicity (Prisco et al., 2025; Cheng et al., 2026). However, current evidence does not directly prove that circadian disruption initiates the intestinal phenotype in PH. A simpler interpretation is also possible: PH-associated hypoxia, congestion, inflammation, or medication exposure may disturb the gut, and circadian disruption may then amplify the injury. Feedback modulation by microbial signals.

The gut microbiota is also a regulator of host timing. SCFAs, especially butyrate, can inhibit histone deacetylases and alter the expression of selected clock-related genes in intestinal epithelial and hepatic cells (Chen et al., 2025c). Bile acid metabolites signal through nuclear receptors such as farnesoid X receptor (FXR), linking microbial metabolism to rhythmic inflammatory and metabolic transcription (Fawad et al., 2022; Prisco et al., 2025). Tryptophan metabolites can influence sleep, neural activity, and immune homeostasis (Dantas MaChado et al., 2022; Hassan et al., 2022). In PH, reduced SCFAs, increased TMAO, and abnormalities in serotonin and kynurenine pathways have been reported (Li et al., 2021; Moutsoglou et al., 2023; Cao et al., 2024; Bravi et al., 2025; Lagod et al., 2025). These observations make microbial metabolites plausible mediators between circadian misalignment and pulmonary vascular disease. Still, plausibility is not proof. Direct evidence that these metabolites reset, blunt, or desynchronize pulmonary vascular clocks in PH remains scarce.

## 3 Roles of circadian dysregulation in PH development and progression

### 3.1 Regulation of PASMCs by core clock genes

**Figure 3:**
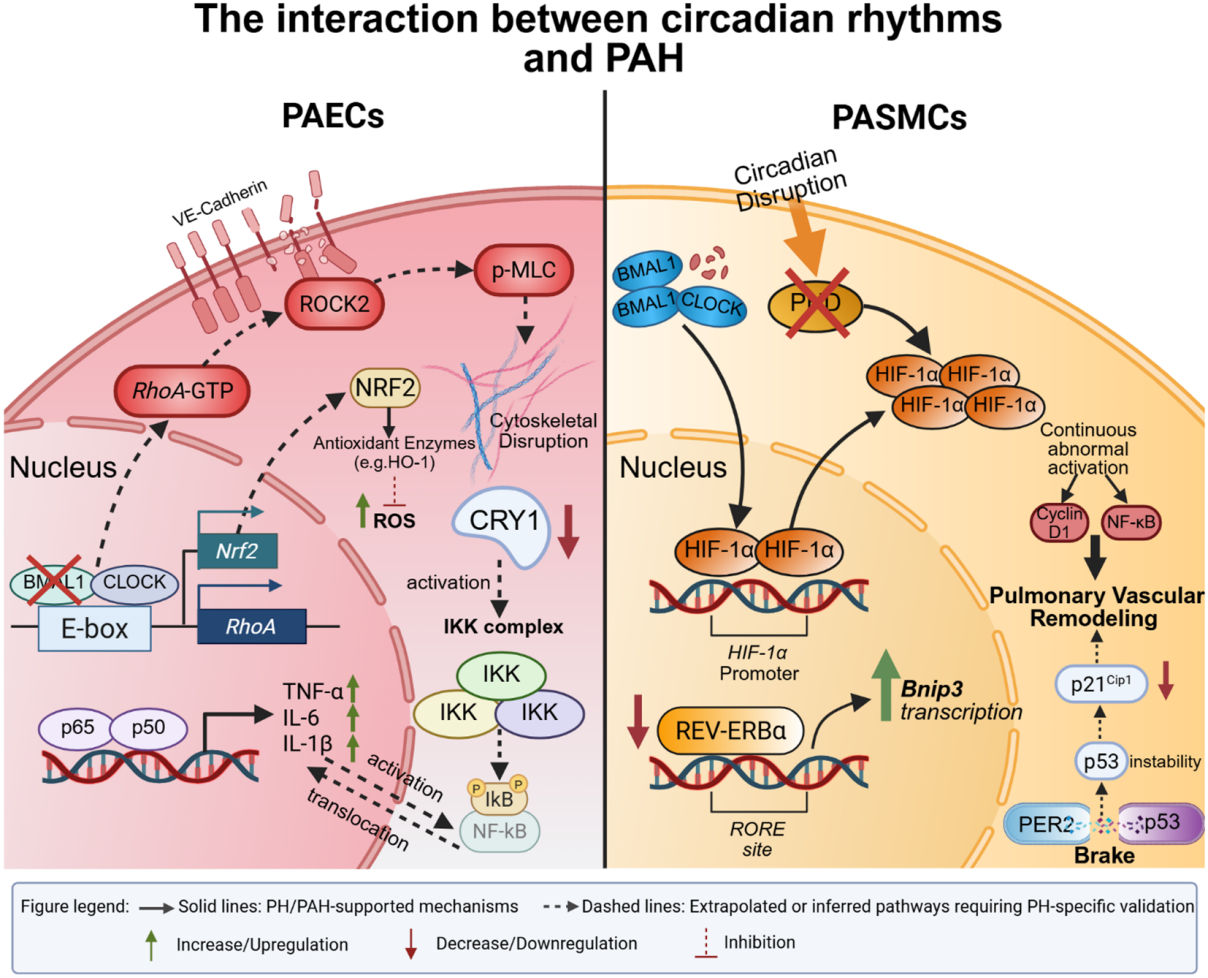
Molecular cross-talk between circadian rhythm disruption and PH pathogenesis. Circadian misalignment may dysregulate cell-specific molecular clocks and thereby contribute to pulmonary vascular remodeling through PH-supported and inferred pathways: BMAL1/CLOCK disruption de-represses RhoA transcription, hyperactivating the RhoA-GTP/ROCK2 axis to phosphorylate MLC, disrupting the cytoskeleton and VE-Cadherin integrity. Concurrently, impaired NRF2 signaling elevates ROS, while CRY1 depletion disinhibits the IKK complex, accelerating NF-κB nuclear translocation and pro-inflammatory cytokine (TNF-α, IL-6, IL-1β) release. Circadian disruption suppresses PHD to stabilize HIF-1α, driving its own transcription and upregulating Cyclin D1/NF-κB. Meanwhile, REV-ERBα downregulation enhances Bnip3 transcription, and PER2-p53 disruption destabilizes p53 to downregulate p21^Cip1^, cooperatively promoting proliferation and remodeling. Solid arrows indicate PH/PAH-supported mechanisms, whereas dashed arrows indicate extrapolated or inferred pathways requiring PH-specific validation. Created using BioRender.com.

Core clock genes not only encode temporal information but also act as transcriptional regulatory hubs in pulmonary vascular cells. By controlling cell-cycle progression, metabolic state, and signal transduction, they may influence the fate of PASMCs and PAECs. PASMC hyperproliferation is a major contributor to medial hypertrophy. Under pathological conditions, disruption of BMAL1 rhythmicity may activate the BMAL1/CLOCK-Cyclin/CDK axis and promote sustained expression of cell-cycle proteins such as cyclin D1 and CDK4, thereby facilitating abnormal PASMC proliferation (Mir et al., 2024). PER2 can interact with and stabilize p53, inducing downstream p21Cip1 expression and maintaining cellular quiescence (Wu et al., 2019). Loss or downregulation of PER2 may weaken this inhibitory checkpoint and promote an apoptosis-resistant phenotype. In addition, the REV-ERBα/BNIP3 axis has been linked to mitophagy and metabolic reprogramming (Imani et al., 2025). In PH, reduced REV-ERBα expression may relieve repression of Bnip3, induce excessive mitophagy, and promote a glycolytic metabolic shift consistent with a Warburg-like phenotype (Imani et al., 2025). BMAL1 may also interact with HIF-1α-related transcriptional programs, and circadian imbalance may favor HIF-dependent phenotypic switching of PASMCs from a contractile toward a synthetic state (Gotoh et al., 2016). Nevertheless, direct evidence for BMAL1-HIF-1α and PER2-related mechanisms in PH remains insufficient (**Figure 3**).

### 3.2 Regulation of PAECs by core clock genes

In PAECs, local circadian clocks may preserve endothelial homeostasis by maintaining intercellular junctions, antioxidant capacity, and inflammatory thresholds. BMAL1 has been associated with endothelial barrier function, VE-cadherin expression, and cell-cycle regulation; CRY proteins may limit excessive inflammatory signaling by modulating NF-κB activity; and the BMAL1-Nrf2/HO-1 axis may contribute to antioxidant defense (Qin and Deng, 2015; Astone et al., 2023; Azzi et al., 2023; Teglas et al., 2023; Dandavate et al., 2024; Teglas et al., 2025; Qiu et al., 2026). When these rhythmic regulatory programs are disturbed by hypoxia, inflammation, or metabolic stress, PAECs may become more susceptible to barrier disruption, oxidative stress, apoptosis, and endothelial-to-mesenchymal transition. Yet the evidence base is thinner than the pathway map suggests. Much of the current literature comes from general vascular endothelium, airway inflammation, or other pulmonary injury models, not from PH-derived PAECs. The next question is therefore specific: do PAECs from PH lungs show reproducible clock amplitude loss, phase shifts, or time-dependent inflammatory sensitization? Without that evidence, endothelial clock dysfunction should be described as a credible mechanism, not a settled conclusion (**Figure 3**).

## 4 Gut-lung axis dysregulation in PH pathophysiology

### 4.1 Gut microbial compositional alterations reported in PH

In recent years, alterations in gut microbiota composition have garnered increasing attention in the context of PH. Evidence from both clinical studies and experimental models suggests that PH-associated dysbiosis commonly involves changes in the Firmicutes-to-Bacteroidetes ratio, depletion of Bacteroidetes or Bacteroides, reduction of short-chain fatty acid-associated taxa, and enrichment of several inflammation-associated or pathobiont-related microbial features, including TMA/TMAO-associated functions. Gut microbial abnormalities have been described in SuHx model, MCT model, chronic hypoxia model, and clinical PAH/CTEPH settings, suggesting that PH-associated gut alterations are not limited to metabolite changes but may also involve bacterial community remodeling, impaired gut barrier function, endotoxin translocation, and systemic inflammatory activation. The major bacterial taxa repeatedly reported to be altered in PH, together with their reported direction, potential biological relevance, and representative references are summarized in **Table 1**.

**Table 1:** Major gut bacterial taxa repeatedly reported to be altered in PH/PAH.

Microbial feature/taxon	Reported alteration	Evidence source	Potential biological significance	Representative references
Firmicutes/Bacteroidetes ratio	Often increased in SuHx and MCT PAH models; direction may differ in chronic hypoxia models.	SuHx rat; MCT rat; hypoxia models; review-level synthesis.	A common dysbiosis marker; may reflect reduced Bacteroidetes, impaired SCFA production, and disturbed immunometabolic homeostasis.	(Callejo et al., 2018; Sanada et al., 2020; Wu et al., 2022; Luo et al., 2023)
Bacteroidetes/Bacteroides	Frequently reduced in SuHx rats and patients with PAH.	SuHx rat; human PAH metagenomics.	Linked to polysaccharide fermentation, propionate/acetate generation, gut barrier protection, and immune homeostasis.	(Callejo et al., 2018; Kim et al., 2020; Sanada et al., 2020)
Akkermansia	Reduced in SuHx rats and patients with PAH.	SuHx rat; human PAH metagenomics.	Associated with mucus-layer integrity, epithelial barrier function, anti-inflammatory effects, and metabolic homeostasis.	(Kim et al., 2020; Sanada et al., 2020; Wu et al., 2022)
Lachnospiraceae	Repeatedly reported as altered; relatively depleted as butyrate-associated taxa in clinical PAH, whereas some members may increase in SuHx models.	Human PAH; SuHx rat; PH microbiome reviews.	Often linked to butyrate production, barrier maintenance, and immune regulation; direction should be interpreted by model context.	(Kim et al., 2020; Sanada et al., 2020; Wu et al., 2022)
Ruminococcaceae/Ruminococcus	Repeatedly reported as altered in SuHx and hypoxia-associated models, with model-dependent direction.	SuHx rat; hypoxia mouse/rat models; high-altitude/hypoxia-related studies.	Involved in fiber degradation, SCFA production, and bile acid metabolism; may link diet, hypoxia, and inflammation.	(Sanada et al., 2020; Wu et al., 2022; Luo et al., 2023)
Prevotella/Prevotellaceae	May increase in SuHx and hypoxia-associated PH models; linked to TMA/TMAO-related functional signatures in clinical PAH.	SuHx rat; hypoxia mouse; human PAH metagenomics.	May be associated with mucosal inflammation, amino-acid metabolism, TMA/TMAO metabolism, and immune activation, but is strongly diet-dependent.	(Kim et al., 2020; Sanada et al., 2020; Wu et al., 2022)
Butyrate- and propionate-producing taxa	Relatively depleted in clinical PAH; SCFA-producing bacteria and acetate levels are reduced in animal models.	Human PAH; SuHx rat; CTEPH inflammatory study.	SCFAs exert anti-inflammatory, barrier-supportive, and immunoregulatory effects; their depletion may promote endotoxin translocation and systemic inflammation.	(Callejo et al., 2018; Kim et al., 2020; Ikubo et al., 2022; Moutsoglou et al., 2023)
Coprococcus/Butyrivibrio/Eubacterium	More abundant in reference subjects, indicating relative depletion in PAH; some related taxa may vary in SuHx models.	Human PAH metagenomics; SuHx rat model.	Typical SCFA-associated taxa, especially linked to butyrate/propionate generation and immune tolerance.	(Kim et al., 2020; Sanada et al., 2020; Wu et al., 2022)
Enterococcus/Enterococcus-related signals	Enterococcus decreased in SuHx rats; Enterococcal phage enrichment reported in human PAH.	SuHx rat; human PAH virome analysis.	Suggests bacterial-virome ecological remodeling; may reflect altered microbial stability and inflammatory milieu.	(Kim et al., 2020; Sanada et al., 2020)
TMA/TMAO-associated microbial functions	Enriched in patients with PAH; involves functional signals related to Clostridium, Prevotella, Streptococcus, Citrobacter, Collinsella, and others.	Human PAH shotgun metagenomics; metabolomic studies.	TMAO may promote inflammation, oxidative stress, endothelial dysfunction, and pulmonary vascular remodeling.	(Kim et al., 2020; Wu et al., 2022; Moutsoglou et al., 2023)

### 4.2 Microbial translocation and imbalance of microbial metabolites

**Figure 4:**
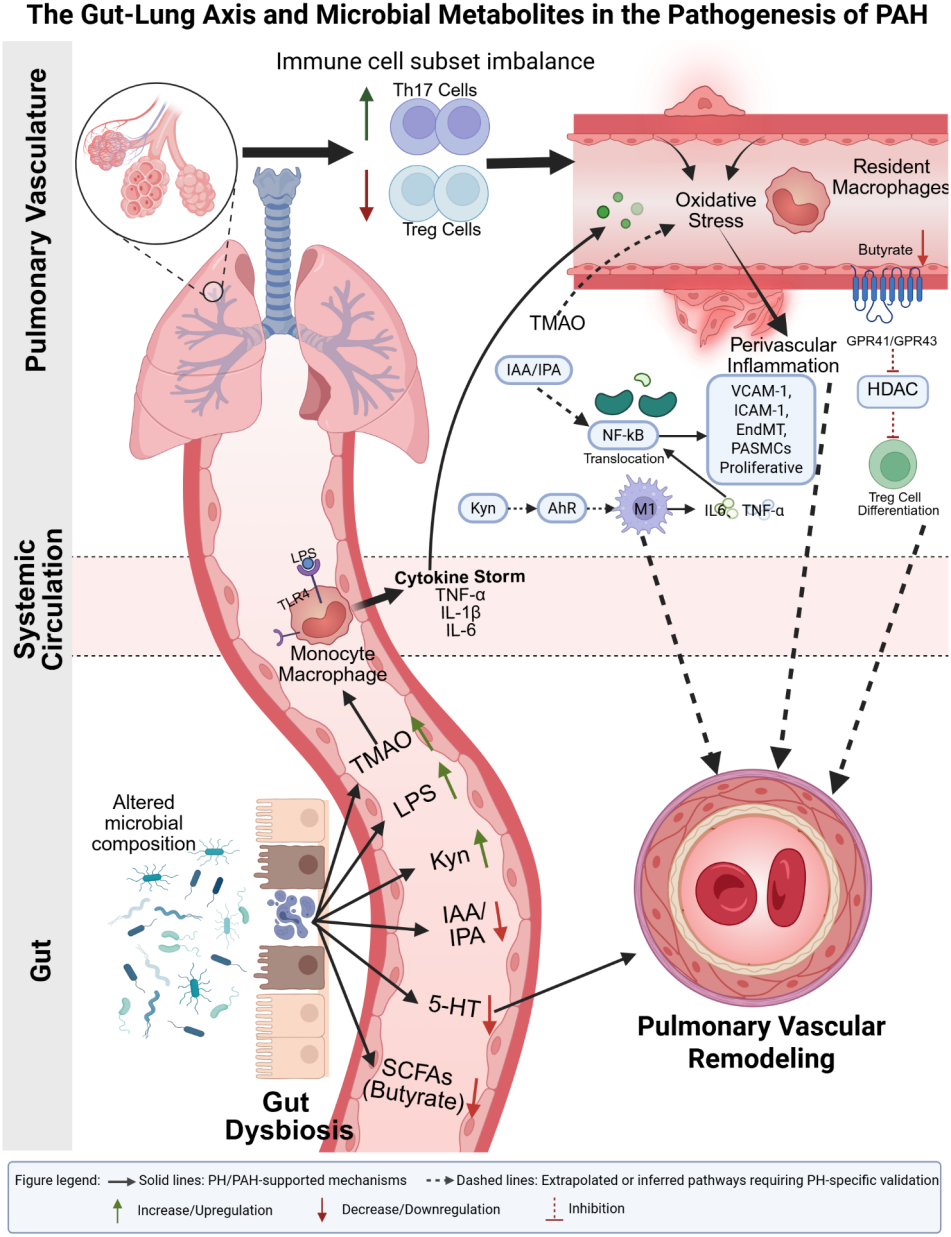
The gut-lung axis and microbial metabolites in PAH pathogenesis. Dysbiosis drives pulmonary remodeling across three compartments. Gut dysbiosis triggers systemic leakage of elevated TMAO, LPS, and kynurenine (Kyn), alongside depleted IAA/IPA and butyrate. Circulating LPS binds TLR4 on macrophages, promoting systemic inflammatory activation involving TNF-α, IL-1β, and IL-6 signaling. Systemic cues disrupt pulmonary homeostasis via three axes: (1) Immune Imbalance: Th17 cell expansion and Treg suppression. (2) Inflammatory Signaling: TMAO elevates oxidative stress; Kyn/AhR polarizes M1 macrophages; depleted IAA/IPA disinhibits NF-κB, driving VCAM-1/ICAM-1 upregulation, EndMT, and PASMC proliferation. (3) Epigenetic Regulation: Butyrate deficiency impairs GPR41/43 signaling, de-repressing HDAC activity to inhibit Treg differentiation and worsen remodeling. Solid arrows indicate PH/PAH-supported mechanisms, whereas dashed arrows indicate extrapolated or inferred pathways requiring PH-specific validation. Created using BioRender.com.

An important mechanism by which gut-lung axis dysfunction may contribute to PH is translocation of microbial-associated molecules resulting from impaired intestinal barrier function. When intestinal epithelial integrity declines, microbial products such as LPS may enter the mesenteric circulation and, after hepatic processing, reach the systemic circulation, inducing low-grade inflammation and immune-cell activation. These inflammatory signals may promote cytokine release, oxidative stress, and vascular inflammation by acting on pulmonary endothelial cells, macrophages, and neutrophils (Maruyama et al., 2025; Yang et al., 2025). A second key mechanism is imbalance of microbial metabolites, particularly reduced SCFAs and altered tryptophan metabolism. SCFAs are produced by beneficial bacteria through dietary fiber fermentation and have anti-inflammatory and barrier-protective effects (Maruyama et al., 2025; Ruan et al., 2025). In PH-related states, a reduction in SCFA-producing bacteria may decrease SCFA availability, weakening intestinal barrier integrity and reducing anti-inflammatory signaling in the lung (Li et al., 2021; Cao et al., 2024). Within the pulmonary environment, SCFAs may promote anti-inflammatory immune polarization through histone deacetylase inhibition and GPR41/43 activation, whereas SCFA depletion may attenuate these protective effects (Fang et al., 2025; Maruyama et al., 2025). Dysregulated tryptophan metabolism may further alter kynurenine and indole derivatives and reshape the pulmonary vascular immune microenvironment (Moutsoglou et al., 2023; Cao et al., 2024; Lagod et al., 2025). Current evidence supports an association between these metabolic changes and PH, but their causal relationships and temporal sequence require confirmation in longitudinal and interventional studies (**Figure 4**).

### 4.3 Trained immunity and monocyte pre-activation

Trained immunity helps explain why a transient gut-derived signal might leave a lasting pulmonary vascular imprint. In this process, monocytes, macrophages, or progenitor cells undergo epigenetic and metabolic reprogramming after an initial stimulus and respond more strongly to later challenges (Li et al., 2025a). Gut-derived LPS, microbial metabolites, and extracellular vesicles can all participate in such immune training (Maruyama et al., 2025). In PH, this concept is useful, but it must be handled carefully. Direct PH evidence remains limited; much of the mechanistic foundation comes from chronic inflammatory disease, cardiovascular disease, and lung injury models (Netea et al., 2016; Cai et al., 2022, 2025; Huang et al., 2025; Shen et al., 2025; Shuai et al., 2025). For that reason, trained immunity is better described as a candidate explanation for persistent gut-lung inflammatory amplification, not as a proven central pathway in PH.

The bone marrow adds another layer. After intestinal barrier disruption, circulating LPS and microbial metabolites may influence hematopoietic stem and progenitor cells, shifting myeloid output and increasing inflammatory readiness (Saaoud et al., 2023; Ruffenach et al., 2024; Akiba et al., 2025; Can et al., 2025; Le et al., 2025; Liao et al., 2025; Otalora-Otalora et al., 2025). Monocytes produced under these conditions could enter the pulmonary circulation already primed, then differentiate into pro-inflammatory macrophages within the pulmonary adventitia and support PASMC proliferation (De Laval et al., 2020; Vanickova et al., 2023; Robles-Vera et al., 2025). PH patients do show altered monocyte maturation and activation states (de Laval et al., 2020), but the complete gut-bone marrow-pulmonary vessel sequence has not been demonstrated in one experimental system. This is an important gap. If validated, it would shift the gut-lung axis from a local microbiome story to a systemic immune-memory model of PH progression.

## 5 Crosstalk between circadian rhythms and the gut-lung axis in PH

### 5.1 Co-regulation of innate immunity and inflammatory response

Circadian rhythms and gut microbiota may converge at the level of innate immune regulation, with the NLRP3 inflammasome representing an important candidate node. BMAL1 and CLOCK regulate immune-cell function and barrier integrity (Ma et al., 2025), while circadian disruption can increase intestinal permeability and LPS exposure (Harper et al., 2025; Kumar et al., 2025). NLRP3 is a key innate immune sensor whose expression and activation exhibit time-dependent regulation and can be influenced by BMAL1/CLOCK signaling (Harper et al., 2025; Hao et al., 2026). Disruption of the molecular clock may facilitate aberrant NLRP3 activation, IL-1β release, pulmonary endothelial injury, and vascular inflammation (Harper et al., 2025; Robles-Vera et al., 2025). SCFAs add a microbial brake to this system by limiting excessive inflammasome activation (Mills et al., 2024; Haacke et al., 2025). The hierarchy of evidence is important: the inflammatory logic is strong, the PH relevance is plausible, but direct time-of-day NLRP3 studies in PH are still lacking. A convincing test would compare NLRP3 activity, LPS exposure, and SCFA levels across circadian phases in PH models.

### 5.2 Spatiotemporal regulation of immune-cell trafficking

The circulating abundance and tissue recruitment of monocytes are time-dependent, providing a temporal perspective for PH inflammation. Monocyte abundance and tissue recruitment vary with sympathetic tone, the bone marrow CXCL12/CXCR4 axis, and cell-autonomous clocks (O'siorain et al., 2024; Chen et al., 2025a; Hua et al., 2025; Sengupta et al., 2025; Tran et al., 2025). Intestinal barrier function and LPS exposure may also vary across the circadian cycle. Under circadian disruption, if the peak release of gut-derived inflammatory signals coincides with peaks in monocyte egress and pulmonary endothelial adhesion molecule expression, pulmonary vascular inflammatory recruitment may be intensified (Schloss et al., 2017; Shi et al., 2025; Wang et al., 2025). This is a sharper hypothesis than simply saying that inflammation is elevated in PH. It predicts phase dependence. Future experiments should therefore collect blood and lung samples at multiple circadian phases and measure monocyte subsets, adhesion molecules, gut-derived inflammatory markers, and pulmonary vascular inflammation together.

### 5.3 Bile acid-mediated liver-gut-lung feedback loop

Bile acid metabolism is an appealing but less proven bridge between the hepatic clock, gut microbiota, and pulmonary vascular function. The rate-limiting enzyme CYP7A1 is rhythmically regulated by BMAL1/CLOCK and REV-ERBα (Heidt et al., 2014; Teppan et al., 2024), while gut microbes further convert primary bile acids into secondary bile acids (Mendez-Ferrer et al., 2008). In the pulmonary vasculature, bile acids may signal through FXR and Takeda G protein-coupled receptor 5 (TGR5). FXR activation can promote endothelial nitric oxide synthase (eNOS)-derived nitric oxide production, suppress NF-κB signaling, reduce inflammatory mediators such as CCL3 and IL-6, and support endothelial homeostasis (Cavalcanti De Albuquerque et al., 2025; Weng et al., 2026). TGR5 activation may increase cyclic AMP, activate protein kinase A, inhibit PASMC proliferation, and help maintain a contractile phenotype (Nguyen et al., 2013; Tran et al., 2025). Circadian misalignment and microbial dysbiosis could theoretically reduce protective secondary bile acids and attenuate FXR/TGR5 signaling, leading to impaired nitric oxide bioavailability, increased endothelin-1, vascular inflammation, and PASMC phenotypic switching (Gao et al., 2014; Jankowski et al., 2025; Sengupta et al., 2025). Although these pathways are biologically plausible, direct evidence in PH remains limited. Future studies should evaluate time-of-day bile acid profiles in PH patients or models and relate them to FXR/TGR5 pathway activity, pulmonary vascular inflammation, and remodeling.

### 5.4 SCFA-mediated epigenetic modulation of the pulmonary clock

SCFAs provide one of the more concrete links between the gut microbiota and host epigenetic timing. Butyrate can inhibit histone deacetylase 3 (HDAC3) and may increase histone acetylation at promoters of selected clock genes, thereby enhancing clock gene transcriptional activity in intestinal and potentially pulmonary vascular cells (Chen et al., 2025). If rhythmic butyrate-producing bacteria are lost, pulmonary vascular cells could receive less periodic HDAC-inhibitory input. In theory, this could blunt BMAL1 or PER2 activity, weaken restraint on c-Myc and cell-cycle programs, and favor PASMC proliferation (Wang et al., 2024; Chen et al., 2025). SCFAs may also act as metabolic zeitgebers by modulating REV-ERBα-related inflammatory and metabolic programs, thereby linking pulmonary vascular metabolic timing to intestinal nutrient processing (Zhang et al., 2018; Imani et al., 2025). This mechanism is attractive because it links diet, microbial metabolism, epigenetics, and vascular remodeling. Yet the key PH experiment has not been done: showing that butyrate restores clock amplitude or reverses pathogenic timing in PH-derived PAECs or PASMCs. Until then, SCFA-mediated clock rescue remains a strong candidate mechanism rather than a therapeutic conclusion.

### 5.5 Diet and feeding time as shared regulators of circadian rhythm and gut microbiota

Dietary composition and feeding time are key shared regulators linking circadian rhythms with the gut microbiota, and they may also represent underrecognized confounders in PH research. Diet supplies substrates for the gut microbiota and shapes the basal state of microbial metabolic pathways, including those related to short-chain fatty acids, bile acids, tryptophan metabolites, and TMA/TMAO (Park et al., 2023; Chen et al., 2024). Feeding time, in contrast, acts as an important synchronizing cue for peripheral clocks and the daily oscillation of the gut microbiota (Zhang et al., 2025b). Classic studies have shown that the gut microbiota fluctuates across the day in both composition and function, with these fluctuations being regulated by host feeding rhythms (Thaiss et al., 2014). High-fat diets and irregular feeding can dampen microbial rhythmicity, whereas time-restricted feeding can partially restore microbial and host metabolic rhythms (Klingbeil et al., 2024; Qiu et al., 2024). In the context of PH, this implies that diet and feeding time may influence pulmonary vascular remodeling by acting in parallel on host circadian organization, microbial community structure, microbial metabolites, and immune-inflammatory status.

### 5.6 The hypoxia-circadian rhythm-gut microbiota circuit

**Figure 5:**
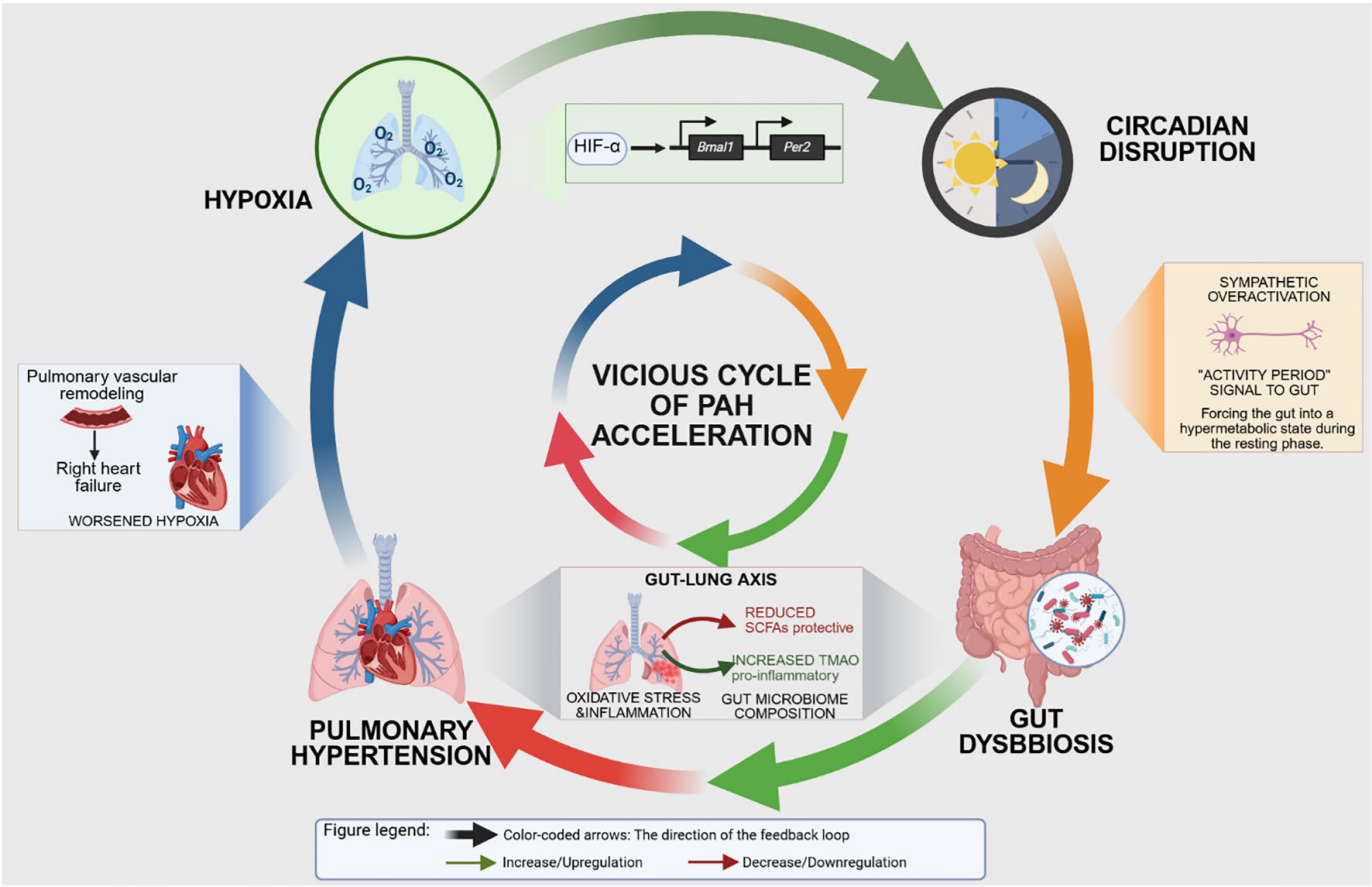
The pathophysiological vicious cycle accelerating PH progression. Chronic disease acceleration is driven by a self-perpetuating, multi-organ feedback loop. Chronic/intermittent hypoxia stabilizes HIF-1α, disrupting core clock gene (*BMAL1/PER2*) transcription. Circadian misalignment enhances sympathetic (SNS) overactivity, forcing the gut into an aberrant hypermetabolic state during its resting phase and driving dysbiosis. Microbiome remodeling depletes protective SCFAs and accumulates pro-inflammatory TMAO, inducing pulmonary oxidative stress and perivascular inflammation via the gut-lung axis. Sustained PH promotes severe vascular remodeling and right heart failure, which further impairs gas exchange, exacerbating hypoxia and fueling the self-perpetuating cycle. Created using BioRender.com.

In PH, chronic hypoxia, circadian disruption, and gut microbial imbalance may form a self-amplifying positive feedback circuit. Chronic hypoxia can act as a disruptive timing signal through HIF-1α-related pathways, suppressing the rhythmic amplitude of core clock genes such as BMAL1 and PER2 and promoting sympathetic activation (Heddes et al., 2022; Zhang et al., 2026). This may place the gut in a metabolically stressed state during the rest phase, contributing to microbial dysbiosis. Dysbiotic microbiota and altered metabolites may then exacerbate systemic inflammation, oxidative stress, and pulmonary endothelial injury through the gut-lung axis, thereby promoting vascular remodeling and worsening hypoxia. As PH progresses toward right heart failure, intestinal congestion and hypoxia may favor the expansion of obligate anaerobes and promote a more pro-inflammatory metabolic profile with reduced rhythmicity, subjecting the pulmonary vasculature to simultaneous hypoxic and gut-derived inflammatory stress (Li et al., 2021; Bello et al., 2024). Thus, the proposed hypoxia-circadian rhythm-gut microbiota circuit begins with hypoxia, disrupts host rhythmicity and gut microbial ecology, accelerates pulmonary vascular remodeling, and thereby feeds back to aggravate hypoxia (**Figure 5**). Based on the available evidence, the hypoxia–circadian rhythm–gut microbiota–pulmonary vascular remodeling feedback loop can be divided into several potential intervention points. These points, however, should be regarded as directions for future experimental validation rather than established therapeutic strategies for PH. First, the upstream hypoxia/HIF–clock interface may provide a chronobiological entry point for testing whether hypoxia disrupts the synchrony between the SCN and peripheral clocks in the lung, gut, liver, and immune system. Second, SCN resynchronization, stable light–dark cycles, sleep–wake rhythm management, and peripheral clock modulation could be used to determine whether restoration of circadian organization attenuates downstream dysbiosis and vascular inflammation. Third, dietary composition and feeding time may serve as non-pharmacological regulatory nodes, as both influence peripheral clock alignment and shape the daily fluctuations of microbial fermentation substrates and gut-derived metabolites. Fourth, the gut microbiota and its metabolites provide more specific intervention targets, including causal testing of microbial alterations, restoration or supplementation of SCFAs, inhibition of the TMA/TMAO pathway, modulation of bile acid–FXR/TGR5 signaling, and intervention in the tryptophan–AhR metabolic axis. Finally, immune-inflammatory nodes should also be examined in a time-dependent manner to determine whether LPS–TLR4 signaling, NLRP3 activation, endothelial adhesion molecules, and monocyte/macrophage recruitment exert stronger pathogenic effects at specific circadian phases. Taken together, these intervention points offer a framework for future studies aimed at interrupting the self-amplifying feedback loop.

## 6 Current evidence gaps and future research priorities

### 6.1 Distinguishing direct PH evidence from extrapolated mechanisms

Although the circadian rhythm–gut microbiota–immune temporal regulation–pulmonary hypertension axis is biologically plausible, the strength of evidence differs across its components. At present, some observations are more directly linked to PH, including gut dysbiosis and altered circulating microbial metabolites in patients with PAH (Kim et al., 2020; Moutsoglou et al., 2023), impaired gut barrier function in experimental PH (Sharma et al., 2020b), activation of inflammatory monocyte/macrophage populations (Florentin et al., 2018), and selected pulmonary vascular remodeling pathways (Florentin et al., 2018). Together, these findings support the involvement of the gut–lung axis and systemic inflammation in PH pathobiology. A second category of evidence comes mainly from PH-related experimental studies or mechanistic work in pulmonary vascular cells. For example, REV-ERBα/BNIP3-associated mitophagy and metabolic reprogramming have been linked to PASMC behavior and vascular remodeling (Qiu et al., 2026), although clinical validation in human PAH remains limited. Similarly, NLRP3 inflammasome activation is closely associated with vascular inflammation (Foley et al., 2021; Al-Qazazi et al., 2022); however, direct evidence that NLRP3 activation in PH is circadian phase-dependent remains limited. These mechanisms should therefore be interpreted as PH-related candidate mechanisms rather than established disease pathways. A third category is extrapolated from non-PH inflammatory diseases, metabolic disorders, cardiovascular disease, or general circadian biology. This category includes CRY1–IKK–NF-κB signaling (Shen et al., 2021), PER2–p53/p21-dependent cell-cycle regulation (Gotoh et al., 2014; Engeland, 2022), SCFA-mediated epigenetic regulation of the clock machinery (Tahara et al., 2018; Fawad et al., 2022), bile acid–FXR/TGR5 signaling (Miyazaki-Anzai et al., 2018), time-dependent leukocyte trafficking (He et al., 2018), and trained immunity (Roring et al., 2025). These pathways provide a biological basis for the proposed model, but their direct contribution to PH needs to be tested in PH-specific experimental systems. To avoid conflating evidence of varying strength into a single tier, we stratified the findings in **Table 2** according to the source of evidence, overall evidence status, current gaps in PH-specific evidence, and priorities for future validation.

**Table 2:** Evidence sources, PH-specific gaps, and future validation priorities for circadian rhythm–gut microbiota–immune timing–PH mechanisms.

Mechanistic node	Direct PH/PAH clinical evidence	PH-relevant experimental evidence	Main non-PH extrapolated evidence	Overall evidence status	PH-specific evidence gap	Future validation priority
Gut dysbiosis	Reported in PH/PAH patient studies	Supported in PH animal models; functional causality remains incomplete	General microbiome dysbiosis literature; IBD/metabolic disease	Partially supported in PH/PAH	Causality, subtype specificity, and temporal sequence remain unclear	Longitudinal PH cohorts; standardized sampling; FMT
SCFAs	Altered metabolites reported; direct PH causal evidence limited	Related PH/inflammation models; anti-inflammatory and epigenetic mechanisms	IBD, immune metabolism, metabolic disease	PH-relevant but largely extrapolated	PH-specific rescue and receptor-level evidence are limited	SCFA supplementation; receptor-specific intervention; time-of-day dosing
TMAO	Partial/limited PH-specific evidence	Limited PH model evidence; vascular and inflammatory effects	Cardiovascular disease, metabolic disease, renal-related literature	Mostly extrapolated to PH	Diet, renal function, and comorbidities confound interpretation	TMAO-pathway inhibition in chronic hypoxia/SuHx PH models
REV-ERBα/BNIP3 axis	Limited human PH clinical relevance	Strong PAH-relevant PASMC/preclinical mechanism	General circadian biology; mitochondrial quality control	Mechanistically plausible; partially PH-relevant	Direct human PH validation remains limited	Time-of-day REV-ERBα modulation; mitophagy and remodeling readouts
NLRP3 inflammasome	Inflammation is clinically relevant in PH; NLRP3-specific clinical evidence partial	Supported in PH/inflammation models; strong innate immune mechanism	IBD, metabolic inflammation, cardiovascular disease	Partially supported in PH; circadian regulation extrapolated	Circadian gating of NLRP3 in PH is insufficiently tested	Time-stamped NLRP3, IL-1β, IL-18 and macrophage profiling
Temporal monocyte trafficking	Partial PH relevance through inflammation/immune remodeling	Limited PH-specific temporal evidence	General immunology; circadian leukocyte trafficking	Largely extrapolated	PH-specific temporal immune mapping is lacking	Circadian immune-cell tracking in blood and lung; adhesion molecule profiling
Bile acids–FXR/TGR5 signaling	Partial PH relevance; direct clinical evidence limited	Limited PH-specific evidence; vascular/metabolic mechanisms plausible	Metabolic disease, liver–gut axis, cardiovascular disease	PH-relevant but largely extrapolated	PH-specific bile-acid causality and receptor effects unclear	Quantitative bile-acid profiling; FXR/TGR5 modulation in PH models
Engineered chronobiotic bacteria	None	No PAH/PH validation; synthetic biology concept	Synthetic biology and chronobiotic design concepts	Speculative/future concept	No direct PH evidence; safety, colonization and rhythm control unresolved	Safety-first animal studies; proof-of-concept before PH efficacy testing
Nanoparticle-mediated butyrate delivery	None	No PAH/PH validation; drug-delivery concept	Nanodelivery, butyrate delivery, drug-delivery biology	Speculative/future concept	No PH-specific delivery, biodistribution or efficacy evidence	Biodistribution, dosing-time, safety and efficacy testing in PH models

### 6.2 PH subtype and model heterogeneity

Another important limitation is the heterogeneity across PH subtypes and experimental models. The mechanisms discussed in this review are most relevant to WHO Group 1 pulmonary arterial hypertension (PAH), and to hypoxia-related experimental PH models, including chronic hypoxia and Sugen/hypoxia (SuHx) models. WHO Group 1 PAH, particularly idiopathic, heritable, and drug-associated PAH, is characterized mainly by pulmonary arteriolar remodeling, endothelial dysfunction, and chronic immune-inflammatory activation (Ferrian et al., 2024; Cober et al., 2025). Clinical studies in PAH have reported distinct gut microbial profiles, depletion of short-chain fatty acid-related taxa, and increased pro-inflammatory microbial metabolic capacity (Kim et al., 2020; Moutsoglou et al., 2023). These findings support a gut–lung and systemic inflammatory component in PAH. By contrast, hypoxia-related PH and chronic hypoxia animal models are more directly driven by sustained or intermittent hypoxic exposure. Hypoxia can activate HIF signaling and pulmonary vascular remodeling (Ma et al., 2023), while also disrupting peripheral clocks, activity–feeding rhythms, and daily oscillations in the gut microbiota (Allaband et al., 2021). PH associated with left heart disease is shaped more by elevated pulmonary venous pressure, fluid retention, intestinal congestion, and heart-failure-related impairment of the gut barrier. Its microbiome evidence is therefore largely extrapolated from heart failure studies and should not be directly equated with PAH (Prisco et al., 2025). CTEPH has a different background, involving thrombosis, vascular remodeling, and coagulation–immune abnormalities (Zhang et al., 2025a). Current microbiome evidence in CTEPH remains limited, and direct mixing with PAH evidence should be avoided. Model selection also matters. The SuHx model combines VEGFR blockade with hypoxic exposure and can produce severe PAH-like pulmonary vascular remodeling (Abe et al., 2010), whereas the monocrotaline (MCT) model has a strong toxic-inflammatory component (Gomez-Arroyo et al., 2012). The timing of inflammatory initiation and microbiome alterations may therefore differ between MCT, SuHx, and chronic hypoxia models.

Future chronobiology studies should therefore be designed around clearly defined PH subtypes and experimental models. Longitudinal sampling should record Zeitgeber time, light–dark conditions, the stage of hypoxic exposure, feeding time, disease stage, and treatment factors, allowing investigators to distinguish whether microbiome alterations act as disease drivers, inflammatory amplifiers, or secondary consequences of disease progression.

### 6.3 Methodological limitations in microbiome and metabolomics studies

Microbiome and metabolome studies provide important support for the presence of a gut-lung axis in PH, but several methodological limitations should be considered. First, many available studies rely on 16S rRNA sequencing. This approach provides useful taxonomic information, but its resolution at the species and strain levels is limited (Johnson et al., 2019). Different strains within the same genus may have distinct metabolic and immunological effects (Duffey et al., 2025). Therefore, an observed change in a bacterial genus cannot be directly interpreted as a change in its functional activity. Second, taxonomic composition does not necessarily reflect microbial function. For example, a reduction in putative SCFA-producing taxa does not by itself prove reduced SCFA production (Frolova et al., 2022). Similarly, detection of a microbial metabolic pathway does not mean that the pathway is active at a given circadian phase. This issue is particularly relevant to the present framework, because the central question is not only whether the gut microbiota is altered in PH, but also whether microbial function and metabolite production lose their normal rhythmicity. In addition, diet and medication are strong covariates that can shape the host metabolome. In patients with PH, diuretics, anticoagulants, PAH-targeted therapies, antibiotics, proton pump inhibitors, and dietary supplements may all act as biological variables that influence microbial composition or metabolite profiles (David et al., 2014; Jackson et al., 2016; Vich Vila et al., 2020).

### 6.4 Testable hypotheses and validation priorities

The hypoxia–circadian rhythm–gut microbiota–pulmonary vascular remodeling loop proposed here should be treated as a set of testable causal hypotheses rather than a descriptive association. Building on the potential intervention points outlined in Section 5.5, future studies should prioritize five questions.

H1: Does hypoxia disrupt host clock synchrony before, or in parallel with, the loss of microbial rhythmicity? Addressing this question will require time-resolved sampling in chronic hypoxia or SuHx models, with integrated assessment of clock genes, the microbiome, the metabolome, and PH phenotypes across lung, gut, liver, blood, and fecal samples.

H2: Does PH-associated dysbiosis have a causal role? This can be tested using fecal microbiota transplantation, antibiotic-mediated microbiota depletion, germ-free animals, or defined microbial consortia, with particular attention to whether the pathogenic effect is enhanced when hypoxia or circadian disruption is present.

H3: Are SCFAs, TMAO, bile acids, and tryptophan metabolites functional mediators rather than static markers? Future experiments should determine whether correction of metabolite imbalance, through metabolite supplementation, pathway inhibition, or receptor modulation, alters PH severity.

H4: Are gut-derived inflammatory signals circadian phase-dependent? This will require measuring LPS–TLR4 signaling, NLRP3 activation, endothelial adhesion molecules, and monocyte/macrophage recruitment at different Zeitgeber times.

H5: Can circadian resynchronization, dietary control, and feeding-time interventions attenuate the feedback loop? These studies should record the light–dark cycle, feeding time, medication timing, sampling time, and PH subtype.

## 7 Research perspectives and cautious translational outlook

### 7.1 Time-resolved multi-omics as a validation framework

**Figure 6:**
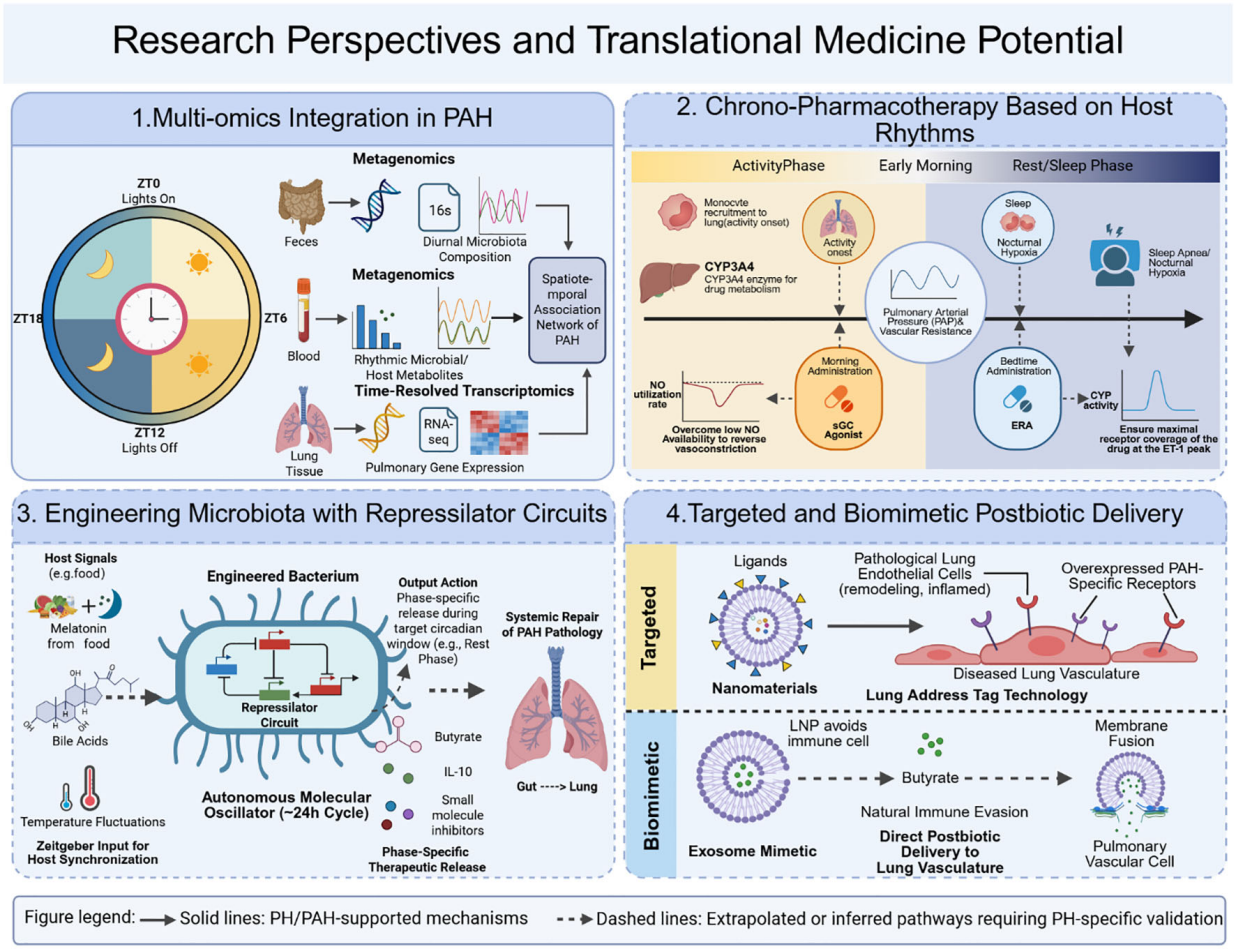
Future research perspectives and validation framework for the circadian rhythm–gut microbiota axis in PH. This schematic summarizes potential research directions rather than established therapeutic strategies. Four areas are highlighted. (1) Multi-omics Integration: Framework combining longitudinal multi-tissue sequencing (ZT0/6/12/18 feces, blood, lung) with metagenomics, metabolomics, and RNA-seq to map dynamic spatiotemporal networks. (2) Chronopharmacology based on host rhythms: future studies may test whether dosing time modifies pharmacokinetics, tolerability, nocturnal hypoxia, vascular tone, or hemodynamic responses to guideline-based PAH therapies. (3) Engineering microbiota with rhythmic circuits: engineered bacteria with autonomous molecular oscillators may provide an experimental platform to examine whether phase-specific release of microbial metabolites or immunomodulatory molecules affects gut–lung communication in PH. (4) Targeted and biomimetic postbiotic delivery: nanomaterials, lipid nanoparticles, or exosome-mimetic vesicles may be explored as preclinical tools to improve lung-directed delivery of microbial metabolites or postbiotics, such as butyrate. Created using BioRender.com.

The most immediate methodological priority is to move toward time-resolved sampling. Static case–control microbiome studies can identify dysbiosis, but they cannot determine whether rhythmicity has been lost or whether microbial and metabolite alterations occur before or after PH progression. In animal models, feces, blood, lung tissue, and right ventricular tissue can be collected at standardized circadian phases, such as ZT0, ZT6, ZT12, and ZT18. In human studies, sampling clock time, sleep–wake status, food intake, medication timing, nocturnal oxygen saturation, and rhythmic markers such as melatonin or cortisol should be recorded. By integrating metagenomics, metabolomics, inflammatory cytokine profiling, and transcriptomics of lung tissue or peripheral blood, future studies could construct a dynamic gut microbiota–metabolite–immune cell–host clock gene network (Li et al., 2008). The key objective should not be limited to identifying differentially abundant taxa. Rather, it should be to determine which microbial communities and metabolites lose circadian rhythmicity, and whether this loss is associated with pulmonary vascular inflammation, right ventricular loading, and disease severity. Such studies should be viewed as a foundation for subsequent causal and interventional work, not as therapeutic strategies in themselves (**Figure 6**).

### 7.2 Chronopharmacology

Chronopharmacology is relevant to PH research because hemodynamics, endothelial function, sympathetic activity, and drug metabolism are unlikely to remain constant over 24 h. Pulmonary arterial pressure, vascular reactivity, endothelin-1, nitric oxide bioavailability, and CYP3A4-dependent metabolism have all been reported to vary across the day in related contexts (Rajagopal et al., 2013; Williams et al., 2023; He et al., 2024; Zheng et al., 2025). Even so, current evidence is insufficient to support changes in PH medication timing in routine practice. A more cautious position is to treat chronopharmacology as a research strategy rather than a clinical recommendation. Prospective studies with strict monitoring could test whether dosing time affects the pharmacokinetics, tolerability, nocturnal oxygenation, morning symptoms, and hemodynamic response of endothelin receptor antagonists, phosphodiesterase type 5 inhibitors, soluble guanylate cyclase stimulators, or prostacyclin analogues. At present, however, medication timing should be considered only as a variable that may refine guideline-based therapy in the future. It should not replace established guideline-recommended treatment (**Figure 6**).

### 7.3 Engineered chronobiotic bacteria

Engineered chronobiotic bacteria represent a long-term concept at the interface of synthetic biology and chronobiology. In principle, tunable oscillatory circuits could be introduced into bacteria to enable time-gated release of butyrate or other beneficial metabolites, thereby mimicking physiological microbial metabolic rhythmicity (Li et al., 2008; Pan et al., 2023; Imakiire et al., 2025). If technically feasible, this approach could provide a tool for studying host rhythmicity and gut–lung axis homeostasis. However, there is currently no direct evidence in PH that engineered rhythmic bacteria can treat disease or reverse pulmonary vascular remodeling. Major translational barriers include stable colonization, synchronization with host rhythms, dose control, ecological safety, biocontainment, and long-term immune effects. Accordingly, this strategy should be presented as a speculative but testable direction for future experiments, rather than a near-term therapeutic option. More precisely, engineered chronobiotic bacteria are currently better suited as an experimental tool to test whether microbial metabolic rhythmicity affects PH biology than as a therapy ready for clinical translation (**Figure 6**).

### 7.4 Inter-organ nanodelivery for epigenetic modulation

Interorgan nanodelivery may offer a potential technical route for increasing effective concentrations of gut-derived metabolites or postbiotics at pulmonary lesions. Butyrate has HDAC-inhibitory activity and may, in theory, affect BMAL1, c-Myc, and cell cycle-related pathways through epigenetic mechanisms. However, oral butyrate is limited by rapid absorption, a short half-life, and insufficient effective concentrations in the pulmonary vasculature (Fawad et al., 2022; Shan et al., 2023). Nanocarriers, lipid nanoparticles, and exosome-mimetic vesicles may improve delivery efficiency, and related technical platforms already exist for lung-targeted nucleic acid or drug delivery (Peek et al., 2017; Pariollaud et al., 2018; Fawad et al., 2022; Qiu et al., 2026; Zhou et al., 2026). Nevertheless, delivery technology should not move ahead of the biology. Before testing nanodelivery of butyrate or other postbiotics in PH, researchers need to define where and when clock disruption occurs within endothelial, smooth muscle, and adventitial compartments, and to determine whether targeted delivery can restore temporal regulation, reduce inflammation, and improve vascular remodeling (Allaband et al., 2021; Moutsoglou et al., 2023). For this reason, nanodelivery should currently be regarded as a preclinical validation platform, not as a therapeutic approach with an established basis for clinical use in PH (**Figure 6**).

## 8 Discussion and conclusion

The circadian system and the gut-lung axis are best understood as parts of a systemic amplification network in PH. They are unlikely to act as isolated primary causes. Current evidence supports a more nuanced interpretation: clock disruption can alter pulmonary vascular cell behavior and, at the same time, weaken intestinal barrier integrity, blunt microbial rhythmicity, shift microbial metabolites, and raise systemic inflammatory tone. PH-related hypoxia, inflammation, and right heart dysfunction can then feed back on host timing and the intestinal ecosystem. This reciprocal structure explains why the disease often extends beyond the pulmonary vessel wall. It also explains why a purely static view of the gut microbiome is incomplete.

The evidence is strongest for gut dysbiosis, altered microbial metabolites, inflammatory monocyte activation, and selected clock-related vascular mechanisms such as REV-ERBα/BNIP3. The evidence is moderate for NLRP3-centered circadian-inflammatory crosstalk and SCFA-mediated epigenetic timing. It is weaker, at least in PH, for bile acid rhythmicity, engineered chronobiotic bacteria, and nanoparticle-mediated butyrate delivery. Recognizing this hierarchy does not weaken the review; it makes the proposed model testable. Future studies should move toward time-stratified longitudinal PH cohorts, paired microbiome-metabolome-immune profiling, nocturnal hypoxia monitoring, and interventional models that manipulate both timing and gut-derived signals. The central question is no longer whether the gut-lung axis is altered in PH. It is whether the timing of that alteration changes the course of pulmonary vascular remodeling.
